# Cancer driver candidate genes *AVL9*, *DENND5A* and *NUPL1* contribute to MDCK cystogenesis

**DOI:** 10.18632/oncoscience.107

**Published:** 2014-12-15

**Authors:** Yaping Li, Jianing Xu, Huan Xiong, Zhongyao Ma, Zhenghe Wang, Edward T. Kipreos, Stephen Dalton, Shaying Zhao

**Affiliations:** ^1^ Department of Biochemistry and Molecular Biology, Institute of Bioinformatics, University of Georgia, Athens; ^2^ Department of Genetics & Genome Sciences and Case Comprehensive Cancer Center, Case Western Reserve University, Cleveland, Ohio; ^3^ Department of Cellular Biology, University of Georgia, Athens; ^4^ Current Address: Human Oncology & Pathogenesis Program, Memorial Sloan-Kettering Cancer Center, New York

**Keywords:** AVL9, DENND5A, NUPL1, MDCK, Cytogenesis, Apicobasal Polarity

## Abstract

*AVL9*, *DENND5A* and *NUPL1* are among the cancer driver candidate genes previously identified via dog-human comparison, and may function in epithelial cell polarity as indicated by bioinformatics analysis. To better understand their cellular functions and roles in cancer, we knocked down each gene in MDCKII cells through shRNA and performed three-dimensional culture. Compared to the control, the knockdown clones developed significantly more abnormal cysts, e.g., cysts with the lumen harboring dead and/or live cells, or cysts having multiple lumens. Further analysis revealed that abnormalities initiated at the first cell division and persisted throughout the entire cystogenesis process. For *NUPL1*-knockdown cells, abnormal cytogenesis largely arose from faulty cell divisions, notably monopolar spindles or spindles with poorly separated poles. For *AVL9*- or *DENND5A*-knockdown cells, abnormalities originated from both aberrant intracellular trafficking and defective mitosis. Moreover, while all knockdown clones displayed an accelerated rate of both cell proliferation and death, only *AVL9*- and *DENND5A*-knockdowns, but not *NUPL1*-knockdown, promoted cell migration. These observations indicate that *NUPL1* contributes to bipolar spindle formation, whereas *AVL9* and *DENND5A* participate in both intracellular trafficking and cell cycle progression. Our study shed lights on these genes' normal cellular functions and on how their alteration contributes to carcinogenesis.

## INTRODUCTION

Via dog-human comparative genomics and oncology studies, we have identified 73 driver candidate genes (likely cancer-causative when altered) and 38 passenger candidate genes (of which alterations are unlikely cancer-causative) for colorectal cancer [1-3]. Bioinformatic analysis indicates that whereas passenger candidate genes' functions appear to be random, driver candidate genes are significantly enriched in functions that are associated with epithelial apicobasal polarity establishment and maintenance [1]. This is consistent with the observation that loss of cell polarity is a hallmark of epithelial cancers [4, 5], and supports the notion that epithelial polarity is a tumor suppressor [4-11].

*AVL9*, *DENND5A* and *NUPL1* are among the 73 driver candidate genes described above. They are deleted in colorectal cancer and restrict cell proliferation of HCT116 and other cancer lines [1]. They are hence classified as putative tumor suppressors. Furthermore, the three genes are annotated to participate in intracellular trafficking that may relate to epithelial cell polarity establishment and maintenance. *NUPL1* encodes nucleoporins p58 and p45 which are components of the nuclear pore complex (NPC) [12], a large transport channel regulating molecular trafficking across the nuclear membrane. DENND5A contains DENN (differentially expressed in neoplastic versus normal cells) domains and interacts with Rab11, Rab6 and Rab39, small GTPases that are indispensable to intracellular membrane trafficking [13-15]. AVL9, having DENN-related AH (Avl9 homology) domains, is an exocytosis gene in yeast [16] and is involved in cell migration [17]. Other than these, no other published studies indicate the involvement of the three genes in cancer or cell polarity.

To experimentally determine if *AVL9*, *DENND5A* and *NUPL1* function in epithelial cell polarity and to better understand how they restrict cancer cell proliferation, we set out to knock down each gene in MDCKII (Madin-Darby canine kidney II) cells, a well-established cell line model for studying cell polarity and epithelial morphogenesis [18-20], and to examine the effects of the gene knockdown on the MDCKII cystogenesis via three dimensional (3D) culture [21].

## RESULTS

### *AVL9*, *DENND5A* and *NUPL1* are knocked down in MDCKII cells through shRNA

**Figure 1:**
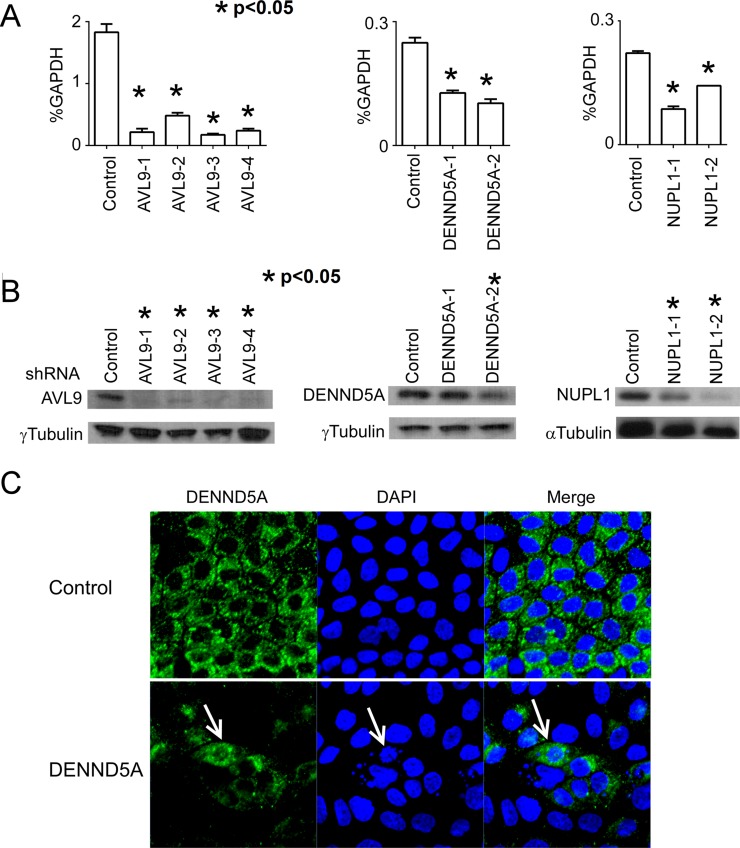
*AVL9*, *DENND5A* and *NUPL1* are knocked down in MDCKII cells via shRNA The control clone is with shRNA against the lacZ gene. The four *AVL9*-knockdown clones are labeled as *AVL9*-1, -2, -3 and -4. Similarly labeled are also the two *DENND5A*-knockdown clones and the two *NUPL1*-knockdown clones. (A and B) The mRNA (A) and protein (B) expression levels of *AVL9*, *DENND5A* and *NUPL1* in each clone were quantified by qRT-PCR and western blot, respectively. The p-values represent the difference in the target gene expression between a knockdown clone and the control, calculated by t-tests with at least three biological replicates. (C) Representative immunostaining images with the anti-DENND5A antibody showing that some abnormal cells (e.g., with fragmentation of the nucleus as pointed by the arrows) have very strong DENND5A expression. This explains why the protein depletion level determined by western blot of *DENND5A*-knockdown clones is insignificant or not as significant as other gene-knockdown clones (B).

Using different shRNA constructs targeting various regions of the *AVL9* gene (Supplementary Table S1), we generated four independent MDCKII clones with stable *AVL9*-knockdown. Meanwhile, we also established two independent MDCKII clones with stable knockdown for each of the *DENND5A* and *NUPL1* genes. Compared to the control, the mRNA reduction rate in the knockdown clones is 70-90% for *AVL9*, ~50-60% for *DENND5A* and 30-60% for *NUPL1* (Figure 1A). At the protein level, the depletion rate is near 90% for AVL9, >50% for NUPL1 and <50% for DENND5A (Figure 1B). Based on our analysis, the lower DENND5A protein depletion rates as determined by western blot experiments do not reflect a failed gene-knockdown, but rather are caused by significantly increased protein expression in certain abnormal/apoptotic cells that emerged because of *DENND5A*-knockdown (Figure 1C). Lastly, the knockdown is well-maintained in each clone, being consistent after 9 cell passages (Supplementary Figure S1). All experiments described below were performed with passage 5 cells.

### *AVL9*, *DENND5A* or *NUPL1* knockdown all alter MDCKII cyst structure

**Figure 2:**
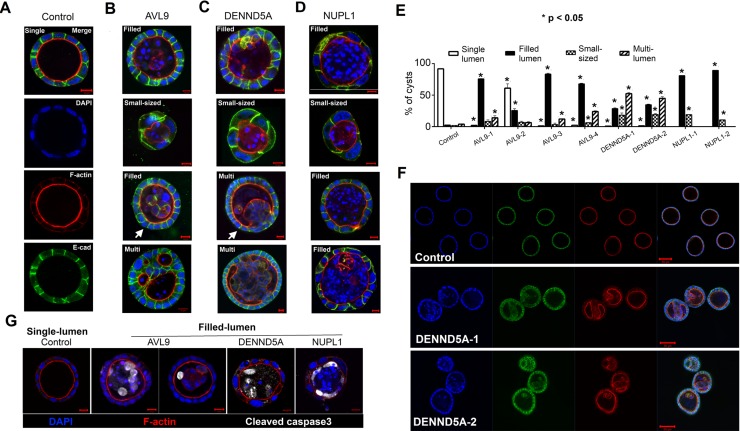
Each gene-knockdown alters cyst structures (A-D) Confocal images of representative cysts of the control (A), and *AVL9-*, *DENND5A*- or *NUPL1*-knockdown clones (B-D) stained for the nucleolus (DAPI), F-actin, and E-cadherin (E-cad). The images represent major cyst types including single-lumen, filled-lumen, small-sized, and multi-lumen. The white arrow in point to the cyst that harbors live cells in its lumen (B), or the cyst that has cells growing into its lumen forming the polyp-like phenotype (C). (E) Percentage of the cyst types illustrated in (A-D) summarized from >100 cysts examined for each clone. Error bars were calculated with three biological repeats. The p-values indicate the difference in the total cyst number of each type between a knockdown clone and the control, calculated by t-tests. (F) An example of confocal images used to compute the data shown in (E). (G) Filled-lumen cysts harbor apoptotic cells in their lumen, as indicated by cleaved caspase3-staining.

To test the hypothesis that each of the three genes plays a role in epithelial morphogenesis as indicated by bioinformatics analysis [1], we took advantage of MDCKII cells' ability to form cysts with apicobasal polarity in 3D culture. Our results show that in our 3D culture environment, >90% of the control cells formed well-organized and approximately equal-sized cysts (Figures 2A, 2E and 2F). Each cyst has a single, cell- or debris-free, and clearly-defined lumen surrounded by a monolayer of epithelia that consist of cells with well-established apicobasal polarity (Figure 2A). This is illustrated with E-cadherin, a cell-cell adherens junction marker that stains the lateral membrane, and F-actin, which nicely outlines the apical region and the lumen (Figure 2A). These well-organized cysts are referred to as “single-lumen cysts” hereafter.

In contrast, *AVL9*-, *DENND5A*- or *NUPL1*-knockdown cells developed mostly aberrant cysts, which could largely be classified into three types: filled-lumen, small-sized, and multi-lumen (Figures 2B-2E). The “filled-lumen cysts” have a single lumen per cyst, but inside the lumen often harbor apoptotic cells (Figure 2G), nuclear fragments and other cellular debris (Figure 2D), and for those of *AVL9*-knockdown clones, sometimes live cells as well (Figure 2B). The “small-sized cysts” are a subset of “filled-lumen cysts” that are notably smaller in size, with the epithelia of each cyst consisting of significantly fewer but larger and often multinuclear cells (Figures 2B-2D). The “multi-lumen cysts” by definition harbor two or more lumens per cyst (Figures 2B and 2C).

### *NUPL1*-knockdown leads to predominantly filled-lumen cysts, while *DENND5A*- or *AVL9*-knockdown results in both filled-lumen and multi-lumen cysts

The distribution of filled-lumen cysts, small-sized cysts, and multi-lumen cysts varies among the gene-knockdowns (Figure 2E). For *NUPL1*-knockdown clones, nearly all cysts are filled-lumen (Supplementary Figure S2), with the lumen harboring apoptotic cells, nuclear fragments and other cellular debris, and with no live cells observed (Figures 2D, 2E and 2G). Other than being filled-lumen, these cysts however appear to retain apicobasal polarity. Indeed, surrounding the lumen is a single layer of cells with F-actin primarily located at the apical side and E-cadherin mostly concentrated at the lateral or sometime basolateral membrane, although F-actin stained more weakly when compared to the control cysts (Figures 2A and 2B). *NUPL1*-knockdown also yielded small-sized cysts, but not multi-lumen cysts (Figure 2E).

Filled-lumen cysts are also frequent for both *AVL*9- and *DENND5A*-knockdown clones. However, a phenotype unique only to *AVL9*-knockdown is that some of its filled-lumen cysts harbor live cells, which even formed cell mass with lumen-facing apical polarity (see the white arrow-pointed cyst in Figure 2B). Another difference is that *AVL9*-knockdown clones developed fewer small-sized cysts, compared to the other two genes (Figure 2E).

Differing from *NUPL1*-knockdown, both *DENND5A*- and *AVL9*-knockdown led to the development of multi-lumen cysts (Figures 2B and 2C). Unlike those in filled-lumen cysts, the cells in certain parts of multi-lumen cysts grew into multiple layers and many cells completely lost their apicobasal polarity (Figures 2B and 2C). Interestingly, multi-lumen cysts appear to have arisen from different mechanisms between the two gene-knockdowns, and we will examine this difference in the next section.

### In control cells, the lumen initiates during the first cell division and expands via polarized cell division

**Figure 3:**
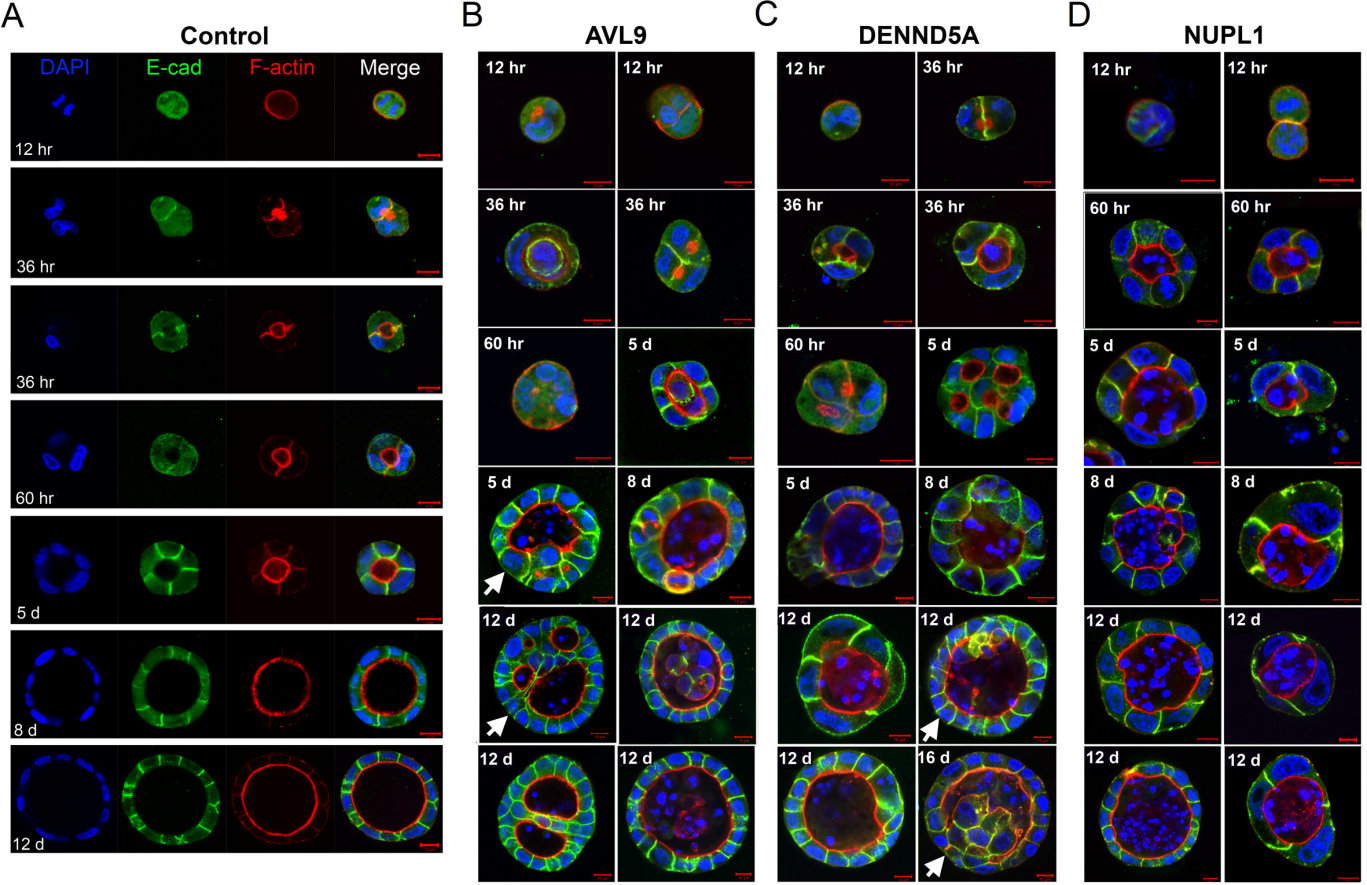
Abnormalities in target gene knockdown clones start at the first cell division and persist throughout the entire cystogenesis process (A) The control clone's time course of cystogenesis. Representative images shows that lumen formation initiates via polarized intracellular trafficking, as indicated by of F-actin and E-cadherin staining, during the first cell division and completes as early as the two- or three-cell stage (also see Supplementary Figure S3). This period is defined as the lumen-establishing stage. Then, the cyst grows in size through orientated cell division (see Figure 4A), and this period is defined as the lumen-enlarging stage. The time course is indicated by hours (hr) or days (d) after the cell-seeding, e.g., “12 hr” and “5 d” representing 12 hours and 5 days after seeding respectively. (B and C) The cystogenesis time course of *AVL9*- or *DENND5A*-knockdown clones. Representative images show abnormalities at the lumen-establishing stage, including no visible lumen-establishment even at the >3 cell stage (the “60 hr” cyst in B), multiple lumen-formation in a single cyst (the “36 hr” cyst in B; the “36 hr” and “60 hr” cysts in C), and off-centered lumen (the left “36 hr” cyst in C). Abnormalities continued at the lumen-enlarging stage, as shown by de novo lumenogenesis (the “5 d” and “12 d' cysts pointed by while arrows in B) and cells actively dividing into the lumen (the “12 d” and “16 d” cysts pointed by white arrows in C). (D) The cystogenesis time course of *NUPL1*-knockdown clone. Note that throughout the cystogenesis, filled-lumen development was widespread but no multi-lumen cysts were observed.

**Figure 4:**
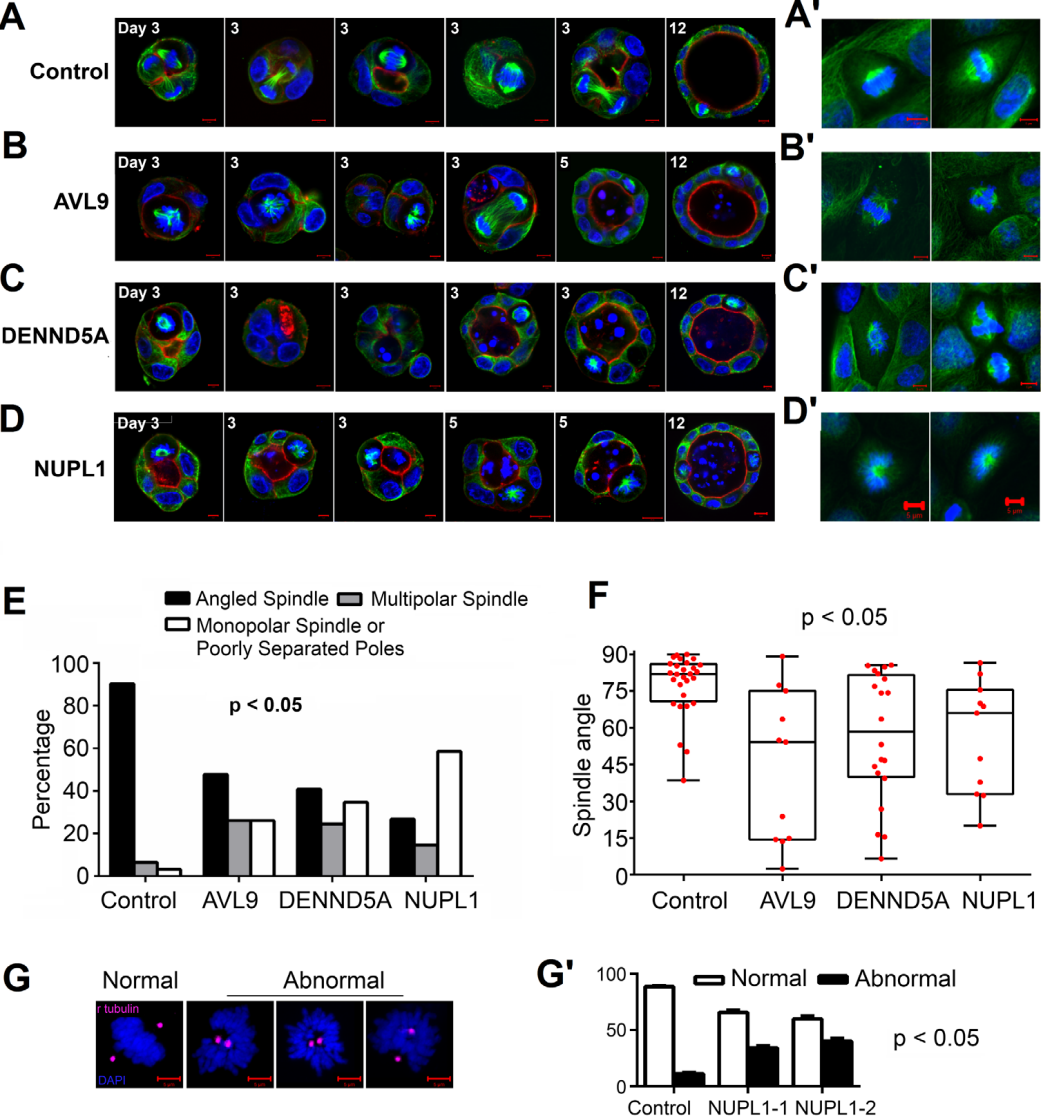
Target gene knockdown clones develop abnormal spindles and other defects (A and A') Control cells' spindles in 3D (A) and 2D (A') culture. Representative images show correctly-oriented spindles and well-organized cell division at both early (day 3) and late (day 12) stages of cystogenesis in control cells. Images of “Day 3” present cell division views from various angles (top, side, center, etc.) of the cyst. (B and B') *AVL9*-knockdown cells developed spindles in 3D culture (B) that are multipolar (1^st^ image), monopolar or with poorly separated poles (2^nd^ and 3^rd^ images), with disorganized microtubules (4^th^ image), or misoriented (5^th^ and 6^th^ images). Multipolar spindles were observed in 2D culture (B'). (C and C') *DENND5A*-knockdown cells in 3D (C) and 2D (C') culture developed similar spindle abnormalities as in (B and B'), along with defects in cytokinesis indicated by the multinucleated in the 2^nd^ image in (C). (D and D') *NUPL1*-knockdown cells developed many monopolar spindles or spindles with poorly separated poles (1^st^ and 4^th^−6^th^ images) (see also Supplementary Figure S4), along with other abnormalities such as multipolar spindles (2^nd^ and 3^rd^ images) in 3D culture (D). Spindles with poorly separated poles were also frequently observed in 2D culture (D'). (E) Statistic summary of different types of abnormal spindles shown in (A-D), compiled from >50 cysts examined for each clone. (F) Spindle orientation for those “Angled Spindles” shown in (E) of the control and target gene knockdown clones. The spindle orientation is indicated by the spindle angle with respect to the radius of the cyst sphere, as described previously [22]. Error bars represent the standard error of the mean. (G and G') γ-tubulin-staining indicates two centrosomes in monopolar spindles or spindles with poorly separated poles developed by *NUPL1*-knockdown cells shown in (D and D'). Representative images of normal and abnormal 2D metaphase cells are shown in (G), and the statistics from at least 30 metaphase cells examined for each clone are shown in (G'). The p-values (all below 0.05) in panels E, F and G' indicate the difference in spindle types (E), angles (F) and kinds (G') as shown between a specified knockdown clone and the control by t-tests.

To better understand spatial-temporal regulation of cystogenesis, we imaged cyst development at different time points. For control cells, cell polarity and lumen formation initiated during cytokinesis of the first cell division (Figure 3A). This was shown by polarized trafficking of F-actin and E-cadherin to the newly established junction between the two daughter cells (Figure 3A). F-actin then congregated around the center of the junction that further developed into the lumen, whereas E-cadherin concentrated along the cell-cell junction that further developed into the lateral plasma membrane compartments (Figure 3A). As early as the two- or three-cell stage, the lumen was established (Figure 3A; Supplementary Figure S3). By day five, nearly all cysts exhibited a single well-defined lumen surrounded by an epithelial monolayer (Figure 3A). Once the lumen was established, the structure of a cyst was maintained and grown through oriented cell division, with the spindle aligned at an approximately 90^o^ angle with respect to the radius of the sphere [22] (Figure 4A). Thus, for the control cells, cystogenesis can be largely divided into three phases: 1) the lumen-establishing stage (seeding to the two or three cell stage; see Figure 3A); 2) the lumen-enlarging stage through orientated cell divisions (primarily from day 3 to day 12; see Figure 3A); and 3) the cyst maintenance stage where most cells cease to divide (after 12 days; not shown in Figure 3A).

### In knockdown cells, abnormalities emerge during the first cell division and persist throughout the entire cystogenesis process

For all knockdown clones, abnormalities start as early as the first cell division and persisted throughout the entire cystogenesis process, affecting both lumen establishment and enlargement as shown in Figures 3B-3D. As elaborated below, abnormalities in cell divisions were observed in all knockdown clones, and in *DENND5A*- and *AVL9*-knockdown clones, deregulated intracellular trafficking were also detected.

Defects in both intracellular trafficking and mitosis were observed in AVL9- or DENND5A-knockdown clones. *AVL9*- or *DENND5A*-knockdown cells harbor a variety of aberrations. In these cells, abnormal lumenogenesis was evident at the lumen-establishing stage, as shown by no visible lumen-establishment even at the >3-cell stage, multiple lumen-formation in a single cyst, or off-centered lumen (Figures 3B and 3C). It appears that polarized intracellular trafficking of F-actin and other molecules, essential for lumen-initiation (Figure 3A), was disrupted. At the lumen-enlarging stage, additional de novo lumenogenesis occurred, resulting in the development of multi-lumen cysts. This is especially common in *AVL9*-knockdown clones (see cysts pointed by white arrows in Figure 3B).

Mitotic defects were also frequently observed in both *AVL9*- and *DENND5A*-knockdown clones. At the lumen-establishing stage, these include failed cytokinesis as shown by multi-nucleated cells, abnormal spindles such as mono- or multi-polar spindles, and disorganized microtubules (Figures 4B, 4C and 4E). These abnormalities continued after the lumen was established. Moreover, misaligned spindles emerged at this phase, with the spindle angles significantly diverging from the normal ~90 degree (Figures 4B, 4C and 4F). Notably, some of these misaligned spindles were otherwise functional and resulted in successful cell division. As a consequence, cells were actively grown into the lumen (see the cysts pointed by the white arrows in Figures 2C and 3C), sometimes yielding multi-lumen cysts (Figures 2C and 2F). This phenomenon was mostly observed in *DENND5A*-knockdown clones.

### Monopolar spindles or spindles with poorly separated poles were frequently seen in *NUPL1*-knockdown cells

The most notable aberration detected in *NUPL1*-knockdown clones in both 3D cytogenesis and 2D culture is monopolar spindles or spindles with poorly separated poles (Figure 4D). This occurred in 60% of spindles examined, significantly higher than other gene-knockdown clones (Figure 4E). Interesting, γ-tubulin staining revealed two centrosomes for many such spindles investigated (Figure 4G). Besides poorly separated spindle poles, a smaller percentage of multipolar spindles and misaligned spindles were also observed (Figures 4E and 4F). However, unlike *DENND5A*- or *AVL9*- knockdown clones, we did not evidently perceive deregulated intracellular trafficking in *NUPL1*-knockdown clones.

### *AVL9* and *DENND5A*, but not *NUPL1*, knockdown promote cell migration

**Figure 5:**
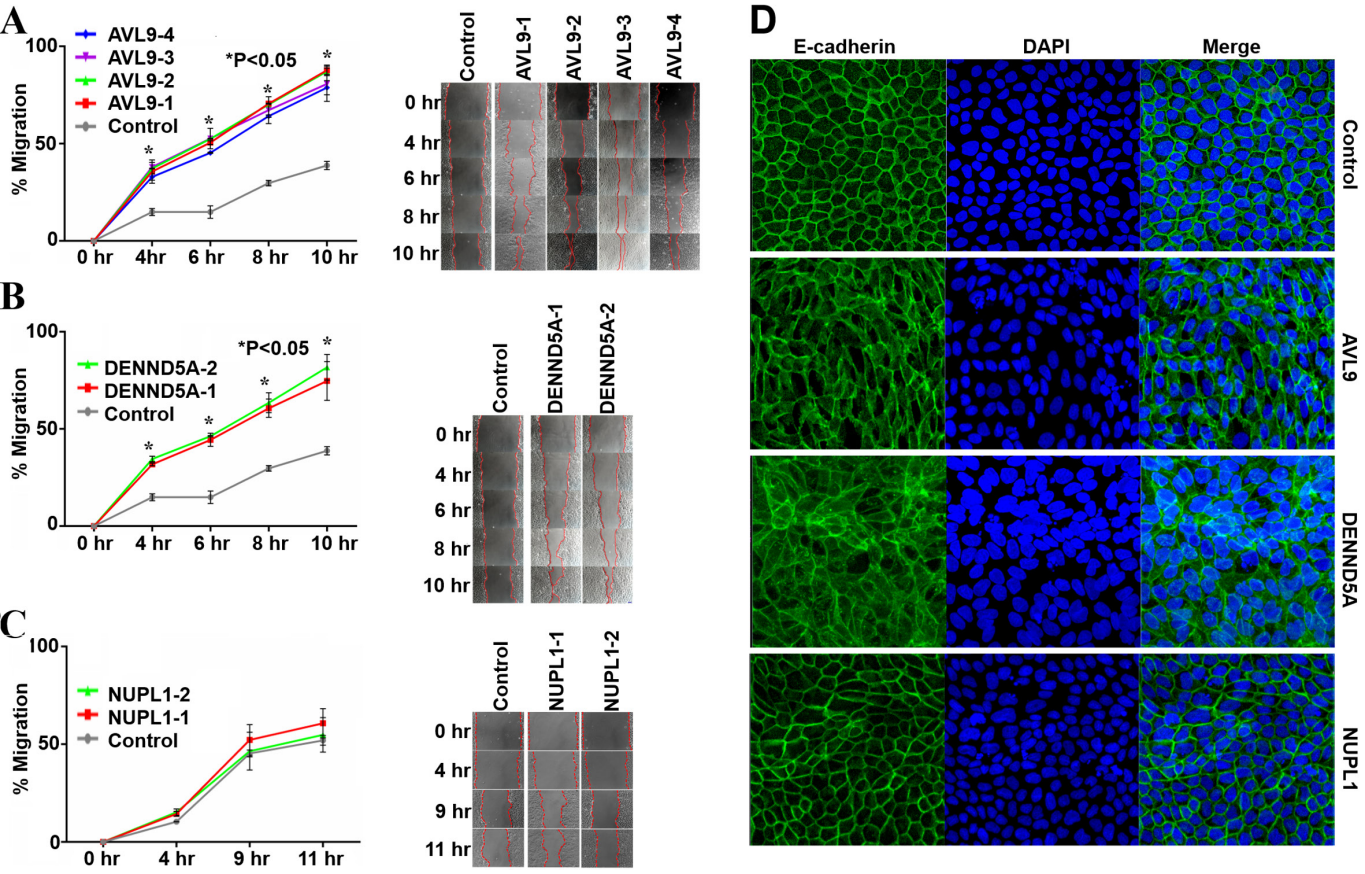
*AVL9*- and *DENND5A*-knockdown, but not *NUPL1*-knockdown, promotes MDKCII migration (A-C) Time course experiments showing the difference in cell migration between the control and the knockdown clones of *AVL9* (A), *DENND5A* (B), or *NUPL1* (C). The plots on the left indicate the cell immigration results at each time point summarized from three biological repeats, one of which is shown in images on the right. The p-value indicate the difference in cell migration between a knockdown clone and the control at an indicated time point, calculated by t-tests from three biological replicates. (D) E-cadherin and DAPI staining of 2D cell culture of the control and target gene knockdown clones as indicated. As revealed by E-cadherin staining, the cell-cell adherens junctions are disrupted in *AVL9-* or *DENND5A-*knockdown cells but are largely preserved in *NUPL1*-knockdown cells, when compared to the control cells.

Compared to the control, all knockdown clones exhibited accelerated cell proliferation accompanied by increased cell death (Figure 3), consistent with previous studies [1]. We further tested whether knockdown of each gene would alter cell migration, another important feature of cancer. We carried out scratch wound healing assays and found that the cell migration rate differed among these gene-knockdowns. While *AVL9*- and *DENND5A*-knockdown clones all migrated significantly faster throughout the time course of the assay than the control cells (Figures 5A and 5B), *NUPL1*-knockdown clones did not (Figure 5C). The finding supports that *AVL9* and *DENND5A*, but not *NUPL1*, play a role in cell migration. It is consistent with the observation that cell-cell adherens junctions, as indicated by E-cadherin staining (Figure 5D), are better preserved in *NUPL1*-knockdown cells than in *AVL9*- or *DENND5A*-knockdown cells (Figure 5D).

## DISCUSSION

Epithelial cells are polarized, with distinct apical and basolateral membrane domain and directional intracellular trafficking (e.g., from ER to Golgi, to endosomes and to plasma membrane). Epithelial morphogenesis requires precise regulation of both cell division and polarity establishment. During carcinogenesis, cell division is deregulated, cell polarity is lost, and epithelial tissue architecture is broken down. We report here that three genes, *AVL9*, *DENND5A* and *NUPL1*, which are recurrently deleted in colorectal cancer [1], all contribute to epithelial morphogenesis.

### *NUPL1* contributes to epithelial morphogenesis by promoting bipolar spindle formation

*NUPL1* encodes nucleoporins p58 and p45, components of the p62 complex that makes up the central channel of the nucleopore, regulating molecular trafficking across the nuclear membrane [12, 23]. During mitosis when the nuclear membrane is disintegrated, *NUPL1* however plays a different role - promoting bipolar spindle assembly, as revealed by our study. *NUPL1*-knockdown leads to the phenotype of monopolar spindles or spindles with poorly separated poles in both 3D and 2D MDCKII cell culture. These abnormal spindles often result in cell cycle arrest and apoptosis [24, 25]. Indeed, throughout the cytogenesis of *NUPL1*-knockdown clones, apoptotic cells have been frequently observed being ejected into the lumen, leading to the widespread development of filled-lumen cysts.

While neither nucleoporin p58 nor p45 has been reported to be involved in cell cycle regulation, other nucleoporins are known to contribute to successful mitosis [26]. Significantly, depletion of Nup98 also leads to monopolar spindle development [27]. The authors have further proposed that Nup98 regulates microtubule dynamics by antagonizing the activity of MCAK (microtubule-depolymerizing mitotic centromere–associated kinesin) to assist bipolar spindle assembly. Whether *NUPL1* also functions through a similar mechanism remains to be determined. Besides deregulated microtubule dynamics, monopolar spindles can also arise from failed centrosome duplication or separation [24]. This mechanism is, however, unlikely in *NUPL1*-knockdown cells, because most of the monopolar spindles do have two centrosomes.

While *NUPL1*-knockdown reduces the apicobasal polarity of the plasma membrane somewhat, it does not completely abolish it. Notably, the cell-cell adherens junctions are largely preserved and *NUPL1*-knockdown has no effects on cell migration. Hence, *NUPL1* is unlikely to be involved in intracellular trafficking that targets adhesion proteins to the plasma membrane, differing from *DENND5A* or *AVL9* described below.

### *DENND5A* and *AVL9* contribute to epithelial morphogenesis by regulating intracellular trafficking and cell cycle progression

In addition to mitotic aberrations such as failed cytokinesis and abnormal spindles, the knockdown of *AVL9* or *DENND5A* has also led to altered intracellular trafficking, disrupted cell-cell adherens junctions and faster cell migration, which have not been observed in *NUPL1*-knockdown cells. Unlike NUPL1, both AVL9 and DENND5A are cytoplasmic proteins. Importantly, DENND5A and possibly AVL9 are regulators of Rab GTPases [15, 17]. DENND5A, also called Rab6IP1 (Rab6-interacting protein 1) in mice, binds Rab6 and GTP-bound Rab11 [28-30] and functions as a GEF toward Rab39 [15, 31]. AVL9 contains DENN-domain related AH regions and is reported to function in the late secretory pathway in yeast [16]. Notably, AVL9-depletion inhibits the migration of A549 human adenocarcinomic alveolar basal epithelial cells [17] (in our study, AVL9-depletion promotes MDCKII cell migration; the difference may be due to different pathways in which AVL9 participates between MDCKII and A549 cells). AVL9 has been proposed to be a GEF for Rab11 or Rab4, although such GEF activity has not been experimentally detected [17].

*AVL9*- and *DENND5A*-knockdown clones in our study share many abnormalities, which may be explained by their common interaction with Rab11 [15, 17, 32] as discussed above. Primarily associated with the pericentriolar recycling endosomes, the Rab11 subfamily members (Rab11a, Rab11b, and Rab25) are critical players in intracellular membrane trafficking [33, 34]. Significantly, a Rab11a-directed network governs de novo generation of apical membrane and lumen in MDCK cells [14, 35, 36]. During the first cell division, apical membrane polarity complex proteins such as Crumbs3a and CDC42 are concentrated in Rab11a-positive recycling endosomes, partitioned between two daughter cells before cytokinesis, and delivered to the site of cytokinesis [14, 36]. This generates a first apical membrane that eventually forms the lumen. Thus, because DENND5A and likely AVL9 interact with Rab11 [15, 17], it is possible that knockdown of either gene perturbs this Rab11a-directed network, resulting in defective lumen establishment during the first cell division. Indeed, the abnormal phenotypes observed during the lumen-establishing stage of *AVL9*- or *DENND5A*-knockdown clones match those of Rab11a-depleted cells [14].

Rab11-dependent membrane trafficking is also required for the completion of cytokinesis [37, 38], and Rab11 has been shown to regulate spindle alignment by modulating metaphase microtubule dynamics in *C. elegans* early embryos [39]. As hypothesized above, these functions are consistent with cytokinesis defects and spindle misalignment observed in *AVL9*- or *DENND5A*-knockdown clones.

Besides the similarities described above, the two gene-knockdowns also differ. First, *DENND5A*-knockdown clones have developed more small-sized cysts. This may be explained by DENND5A's additional interactions with Rab6, which regulates vesicular trafficking within the Golgi and post-Golgi compartments [32, 40, 41]. Importantly, Rab6A' (one of the isoforms) has been proposed to regulate the metaphase/anaphase transition by inactivating Mad2-spindle checkpoint through its regulators/effectors [42], and Rab6A' alteration blocks cells in metaphase [43]. Thus, it is possible that *DENND5A*-knockdown alters the function of Rab6A', blocking the cells in metaphase which leads to the development of multinucleated cells and small-sized cysts.

Significantly, *DENND5A*-knockdown yields cysts with cells actively growing into the lumen, resulting in a phenotype resembling human colonic polyps. This observation supports the tumor suppressing role of DENND5A and is consistent with other DENN domain-containing proteins such as DENND2D, which is proposed to be a tumor suppressor of gastric cancer [44]. How the polyp-like phenotype develops in *DENND5A*-knockdown cells needs further studies. Is it because of spindle misorientation alone or in combination with other mechanisms that make the cells reenter the cell cycle? Lastly, the protein expression of DENND5A has increased sharply in certain abnormal and apoptotic cells. The underlying mechanism and the significance of this finding require further studies.

Clearly, more studies are required to understand the molecular mechanisms through which *DENND5A* and *AVL9* contribute to MDCKII cystogenesis. To investigate the relationship between each gene and the Rab GTPases discussed above may yield valuable insights into the mechanisms.

### Our study supports the notion that epithelial polarity is a tumor suppressor

Epithelia such as those from the colon are highly organized tissues consisting of a single layer of cells with apicobasal polarity. While loss of cell polarity and break down of tissue architecture are hallmarks of epithelial cancers, whether they are drivers or passengers of cancer is however less clear [4-11]. Our work reported here supports that they are cancer drivers, as polarized epithelial tissue architecture itself functions as a non-cell-autonomous tumor suppressor as hypothesized below.

**Figure 6:**
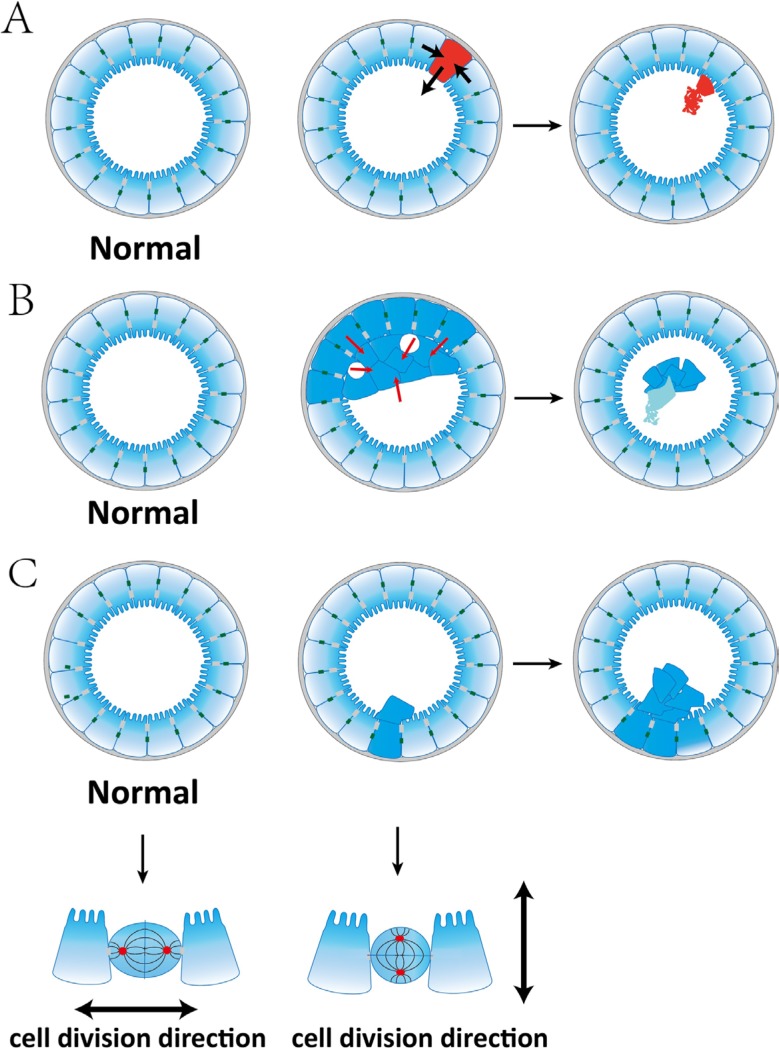
Polarized epithelial tissue architecture suppresses tumorigenesis through three hypothesized mechanisms (A) Epithelial tissue architecture as a whole serves as a non-cell-autonomous tumor suppressor by pushing mutant cells into the lumen. The mutant cell is shown in red and will undergo apoptosis, as illustrated by the development of filled-lumen cysts shown in Figure 2G. (B) Epithelial tissue architecture as a whole serves as a non-cell-autonomous tumor suppressor by releasing abnormal cells into the lumen through lumen-merge. A multi-lumen cyst (e.g., the cyst shown in the last image of Figure 2B) could evolve to a filled-lumen cyst (e.g., the cyst pointed by white arrow in Figure 2B) by merging the lumens and releasing the often multi-layered and not apicobasal-polarized cell mass (indicated by red arrows) into the lumen. The cell mass will eventually undergo apoptosis. (C) Spindle orientation maintains epithelial monolayer and suppresses tumorigenesis. Misorientated spindles lead to cells dividing into the lumen, forming polyps (see the cyst pointed by a white arrow in Figure 2C).

First, those filled-lumen cysts developed in the knockdown clones nicely illustrate that epithelial tissue architecture as whole can serve as a non-cell-autonomous tumor suppressor, as previously hypothesized [5, 6]. The knockdown of each gene results in cell cycle defects, yielding cells carrying additional abnormal changes. Many such cells are then being ejected to the lumen, possibly through mechanisms such as “purse string” wound-healing [6], and are disintegrated through apoptosis (Figure 6A). Eliminating these cells better maintains the integrity of the cyst and allows the cyst to grow. Indeed, the filled-lumen cysts in *AVL9*-knockdown cells become increasing more normal-looking (e.g., in apicobasal polarity) at later stages of cystogenesis. It is possible that mutant cells are also eliminated similarly (Figure 6A) in the colon and rectum, suppressing colorectal tumorigenesis.

Some filled-lumen cysts of *AVL9*-knockdown clones harbor live cells. We hypothesize that these cysts are actually evolved from multi-lumen cysts that have emerged earlier. As a multi-lumen cyst develops, the lumens enlarge and merge at certain points, pushing the often multi-layered and not apicobasal-polarized cell mass into the lumen (Figure 6B). The cell mass is then no longer attached to any matrix and will eventually undergo apoptosis. Thus, this process can also eliminate abnormal cells from the system (Figure 6B), which in our hypothesis provides another mechanism for polarized epithelial architecture functioning as a tumor suppressor.

Finally, correctly orientated spindles maintain the epithelial monolayer [22], directly suppressing tumorigenesis (Figure 6C). To the contrary, misorientated spindles lead to cells dividing into the lumen, forming polyps (Figure 6C). This is possibly a mechanism for the development of the polyp-like phenotype in *DENND5A*-knockdown cells.

More studies are clearly required to test these hypotheses (Figure 6) and to understand the molecular pathways through which polarized epithelial tissue architecture suppresses tumorigenesis in a non-cell-autonomous fashion. The importance of non-cell-autonomous regulations in cancer has been elegantly illustrated by a recent publication [45].

## Methods

### MDCKII cell culture

MDCKII cells, kindly provided by Dr. Karl Matlin of University of Chicago, were grown in Eagle's minimal essential medium (MEM) with 5% (v/v) fetal bovine serum (FBS), 100 U/ml penicillin, 100 mg/ml streptomycin, 10 mM HEPES and 2 mM Glutamine. For 3D culture, MDCKII cells were grown in matrigel, either embedded or on-top, to develop cysts as described [14].

### Antibodies and fluorescent dyes

Antibodies used include anti-E-cadherin (Santa Cruz, Dallas, Texas, USA), anti-α-tubulin (DM1A clone from Sigma, Saint Louis, MO, USA), anti-γ-tubulin (Abcam, Cambridge, MA), anti-cleaved caspase3 (Cell Signaling, Danvers, MA, USA), anti-NUPL1 (Sigma), anti-DENND5A (Abcam), and anti-AVL9 (Abcam). Alexa Fluor^®^ 488 or 647 conjugated secondary antibodies are from Jackson Immunoresearch, West Grove, PA, USA. Rhodamine phalloidin for F-actin-staining and DAPI (1:1000) for nucleus-staining are from Invitrogen, Grand Island, NY, USA.

### Immunofluorescence and confocal microscopy

MDCKII cysts from 3D cell culture were immunostained by indirect immunofluorescence as described [46]. Briefly, the cysts in matrix were fixed with 4% PFA, permeablized with permeablization solution (1× PBS with 0.5% Triton X-100) and blocked with blocking solution (1x PBS with 10% FBS, 3% bovine serum albumin, 0.2% Triton X-100, and 0.2% Tween-20). The cysts were then hybridized with primary antibodies at 4°C overnight followed by secondary antibodies (and DAPI for nuclear staining and rhodamine phalloidin for F-actin staining when needed) at room temperature for 2-3 hours. Finally, the mounting solution, Fluoro-Gel from Electron Microscopy Sciences, was added, and images were taken with a Zeiss LSM 710 confocal microscope.

### shRNA plasmid constructs

shRNA plasmids were constructed using the pRNAi-H1-puro vector system (Biosettia, San Diego, CA, USA) per the manufacturer's instruction. Briefly, the oligonucleotide encoding shRNA sequences (see Supplementary Table S1) were cloned into the pRNAi-H1-Puro vector, which contains the H1 RNA polymerase III promoter and the puromycin marker. The cloned plasmids were confirmed by Sanger sequencing. The plasmid containing oligonucleotide encoding shRNA against the lacZ gene was provided by the company (Biosettia, San Diego, CA, USA) and used as the control.

### Nucleofection and selection of stable knockdown clones

The shRNA plasmids were linearized by *Pac*I and introduced into the MDCKII cells by nucleofection using the Amaxa Cell Line Nucleofector Kit L (Lonza, Allendale, NJ, USA), following the manufacturer's instructions. Transfected cells were selected by growing in medium containing 4μg/mL puromycin until >99% cells in the mock-infected wells were killed. Single colonies were then picked and expanded. At the same time, RNA from the subculture of the cells was extracted and the knockdown efficiency was evaluated by qRT-PCR. The stable knockdown clones were maintained in the presence of 4μg/mL puromycin.

### Migration assay

The scratch-wound cell migration assay was carried out as described [17]. Briefly, more than 1 × 10^5^ of control and knockdown cells were plated in triplicate in 24-well plates. Twenty four hours later after reaching >90% confluency, the growth media were replaced with those without FBS. Twelve hours later, wounds were scratched in the monolayer using a 200 μl pipette tip, and pictures were taken at different time points after the scratch.

## SUPPLEMENTARY TABLE AND FIGURES


