# Supplementary material for: Cancer driver candidate genes AVL9, DENND5A and NUPL1 contribute to MDCK cystogenesis
Source: Oncoscience. 2014 Dec 15;1(12):854–65. doi: 10.18632/oncoscience.107 (PMC4303893; doi:10.18632/oncoscience.107)
Supplement: Supplementary file 1 [file oncoscience-01-0854-s001.pdf]

Cancer driver candidate genes *AVL9, DENND5A* and *NUPL1* contribute to MDCK cystogenesis

Supplementary Materials

Table S1: RNAi sequences used in the study

AVL9-1	GCTCCTGACATTTCAAATACC
AVL9-2	GCAAAGGACAAGAACCCAATG
AVL9-3 and *AVL9*-4	GGATATCTGTGTTTGCCTTAT
DENND5A-1 and *DENND5A*-2	GCATGTCTATGTCCCTATTCT
NUPL1-1 and *NUPL1*-2	GCAGTACAGACAGCAGATTGA

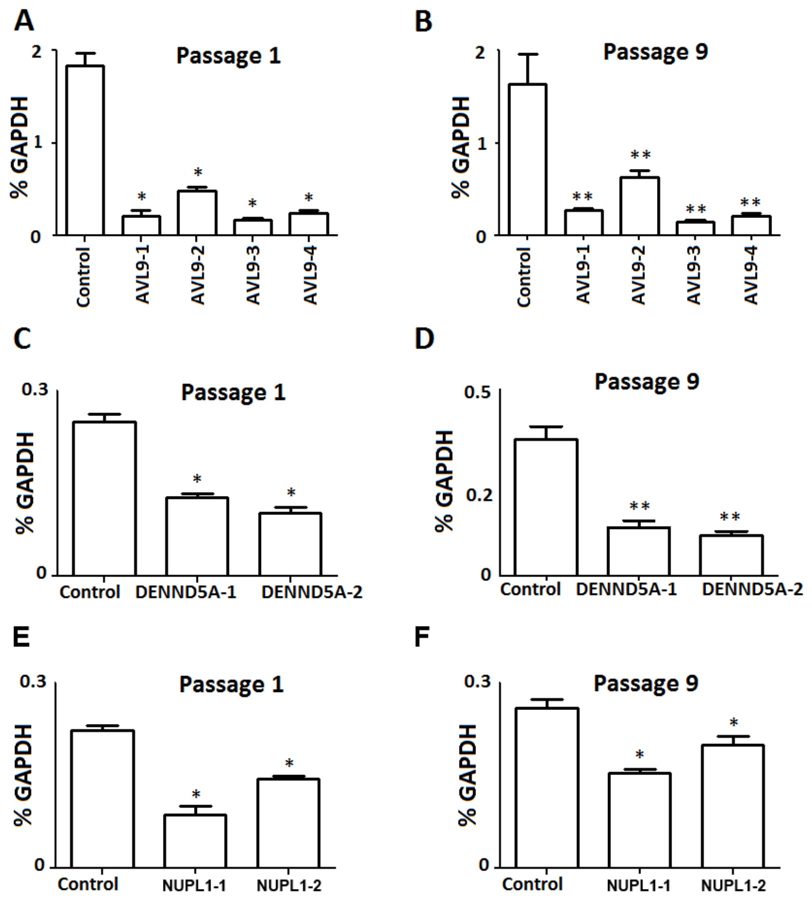

Figure S1: Control and target gene knockdown in MDCKII cells. The mRNA expression level of *AVL9* (**A** and **B**), *DENND5A* (**C** and **D**), and *NUPL1* (**E** and **F**) in the control and knockdown clones at passage 1 or 9 was quantified by qRT-PCR. The p-values (**: $p < 0.05$ and *: $p < 0.01$) represent the difference in the target gene expression between each knockdown clone and the control, calculated by t-tests with at least three biological replicates.

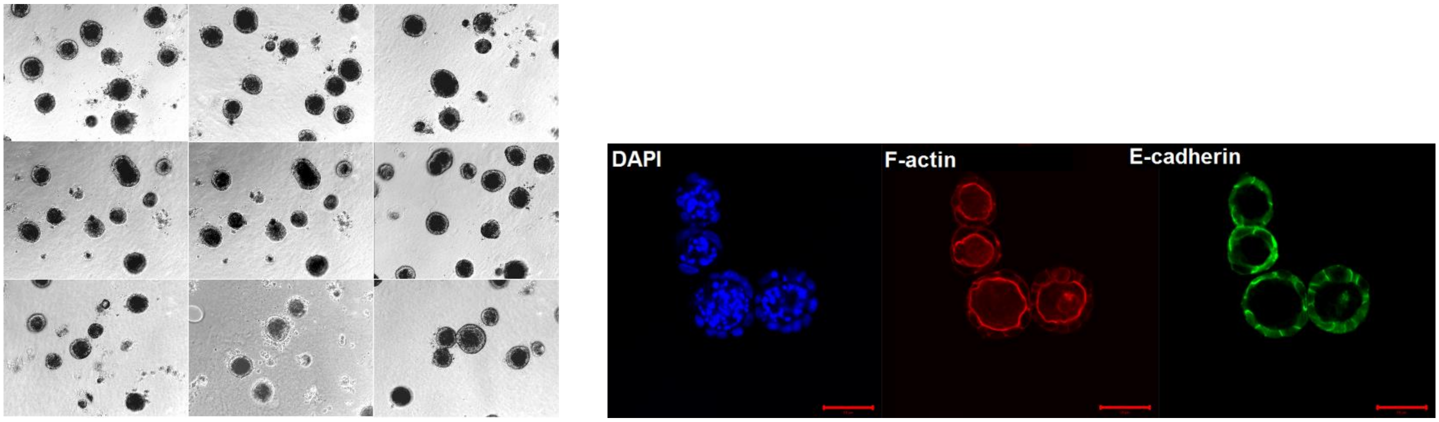

Figure S2: The cysts developed by *NUPL1*-knockdown clones are nearly all filled-lumen, as shown by live cell images (left) and confocal fluorescence imaging (right).

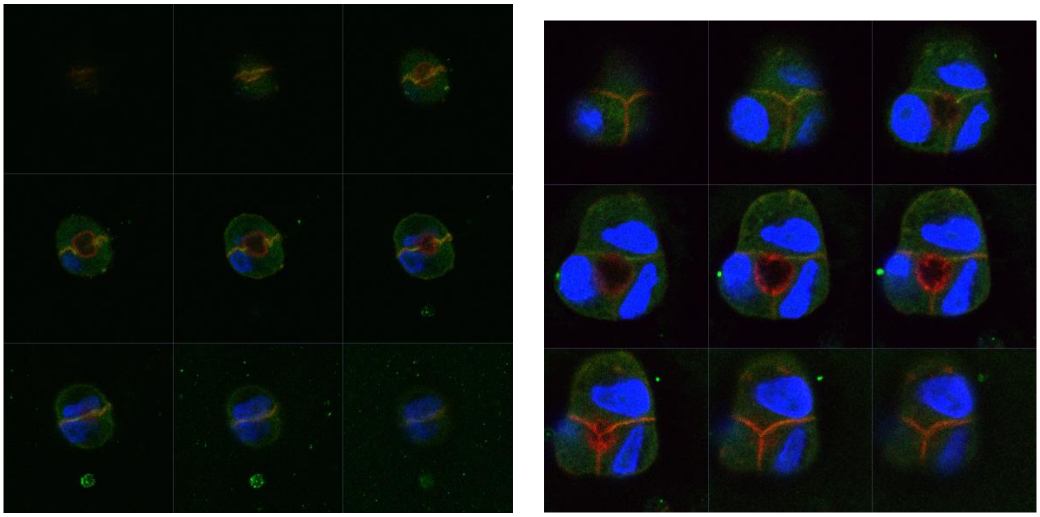

Figure S3: Confocal z-stack images indicate that in control cells, the lumen is established as early as the two-cell (left) or three-cell stage.

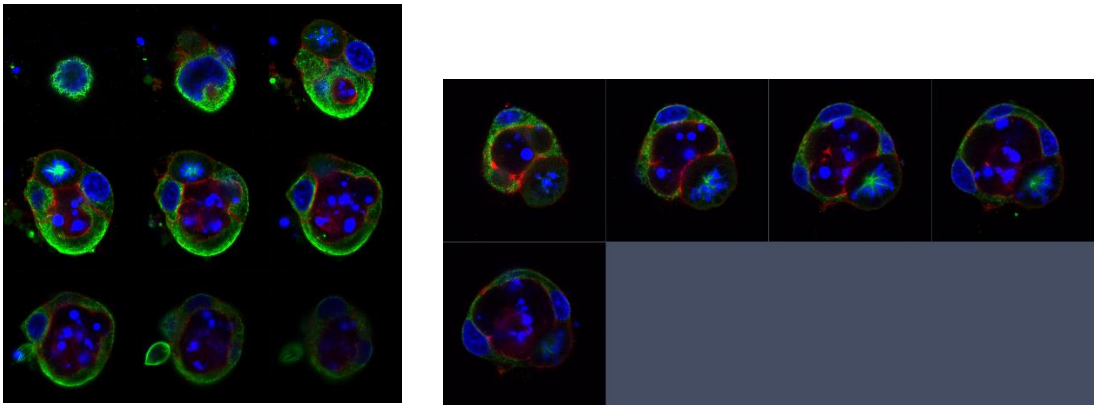

Figure S4: Confocal z-stack images showing monopolar spindles or spindles with poorly separated poles developed by *NUPL1*-knockdown cells.
